# Plant traits and associated data from a warming experiment, a seabird colony, and along elevation in Svalbard

**DOI:** 10.1038/s41597-023-02467-7

**Published:** 2023-09-04

**Authors:** Vigdis Vandvik, Aud H. Halbritter, Inge H. J. Althuizen, Casper T. Christiansen, Jonathan J. Henn, Ingibjörg Svala Jónsdóttir, Kari Klanderud, Marc Macias-Fauria, Yadvinder Malhi, Brian Salvin Maitner, Sean Michaletz, Ruben E. Roos, Richard J. Telford, Polly Bass, Katrín Björnsdóttir, Lucely Lucero Vilca Bustamante, Adam Chmurzynski, Shuli Chen, Siri Vatsø Haugum, Julia Kemppinen, Kai Lepley, Yaoqi Li, Mary Linabury, Ilaíne Silveira Matos, Barbara M. Neto-Bradley, Molly Ng, Pekka Niittynen, Silje Östman, Karolína Pánková, Nina Roth, Matiss Castorena, Marcus Spiegel, Eleanor Thomson, Alexander Sæle Vågenes, Brian J. Enquist

**Affiliations:** 1 Department of Biological Sciences, University of Bergen, Bergen, Norway; 2 Bjerknes Centre for Climate Research, University of Bergen, Bergen, Norway; 3 NORCE, Norwegian Research Centre AS, Bjerknes Centre for Climate Research, Bergen, Norway; 4 Department of Biology, University of Copenhagen, Copenhagen, Denmark; 5 Institute of Arctic and Alpine Research, University of Colorado Boulder, Boulder, USA; 6 Life- and Environmental Sciences, University of Iceland, Reykjavík, Iceland; 7 Faculty of Environmental Sciences and Natural Resource Management, Norwegian University of Life Sciences, Ås, Norway; 8 School of Geography and the Environment, University of Oxford, Oxford, UK; 9 Department of Ecology and Evolutionary Biology, University of Arizona, Tucson, USA; 10 Department of Botany, University of British Columbia, Vancouver, Canada; 11 Department of Ethnobotany, University of Alaska, Fairbanks, Canada; 12 Universidad Nacional de San Antonio Abad del Cusco, Cusco, Perú; 13 Geography Research Unit, University of Oulu, Oulu, Finland; 14 School of Geography, Development and Environment, University of Arizona, Tucson, USA; 15 Department of Health and Environmental Sciences, Xi’an Jiaotong-Liverpool University, Suzhou, China; 16 Department of Biology, Colorado State University, Fort Collins, USA; 17 Department of Environmental Science Policy and Management, University of California, Berkeley, Berkeley, USA; 18 Department of Plant Sciences, University of Cambridge, Cambridge, United Kingdom; 19 Section of Botany, Carnegie Museum of Natural History, Pittsburgh, USA; 20 Department of Botany, Charles University, Prague, Czech Republic; 21 Department of Physical Geography, Stockholm University, Stockholm, Sweden

**Keywords:** Climate-change ecology, Ecosystem ecology, Carbon cycle, Biodiversity

## Abstract

The Arctic is warming at a rate four times the global average, while also being exposed to other global environmental changes, resulting in widespread vegetation and ecosystem change. Integrating functional trait-based approaches with multi-level vegetation, ecosystem, and landscape data enables a holistic understanding of the drivers and consequences of these changes. In two High Arctic study systems near Longyearbyen, Svalbard, a 20-year ITEX warming experiment and elevational gradients with and without nutrient input from nesting seabirds, we collected data on vegetation composition and structure, plant functional traits, ecosystem fluxes, multispectral remote sensing, and microclimate. The dataset contains 1,962 plant records and 16,160 trait measurements from 34 vascular plant taxa, for 9 of which these are the first published trait data. By integrating these comprehensive data, we bridge knowledge gaps and expand trait data coverage, including on intraspecific trait variation. These data can offer insights into ecosystem functioning and provide baselines to assess climate and environmental change impacts. Such knowledge is crucial for effective conservation and management in these vulnerable regions.

## Background & Summary

Arctic regions are currently warming at rates four times the global average^1,2^ while also being affected by other global environmental changes, such as the ongoing loss of seabird populations, which have declined by more than 70% since the 1950s^3,4^. Despite the substantial magnitude and impact of global changes in polar regions, biodiversity and ecosystems do not always follow suit. For example, High Arctic sites are often reported to be relatively resistant to both climate and environmental change^5–7^. Variable ecosystem responses to rapid environmental changes across the Arctic biome call for integrated assessments to understand variations in the magnitude of global environmental change drivers, processes underlying the responses of Arctic vegetation, the consequences of these environmental and vegetation changes for ecosystem functioning, and potential feedbacks to the climate system^8^.

Because the primary productivity of Arctic vegetation is generally temperature-limited, climatic warming has the potential to substantially impact the biodiversity, structure, and functioning of this unique and characteristic biome. Accordingly, widespread vegetation changes are being reported across the Arctic, including advancing phenologies, species range shifts, shrubification, shifts in plant community composition and productivity, and associated changes in ecosystem carbon, nutrient, and water fluxes^9–12^. These widespread vegetation changes emphasize the urgent need to understand and characterize the intricate responses of Arctic ecosystems to ongoing climate change. Seabirds play important roles as ‘ecosystem engineers’ of terrestrial ecosystems on islands worldwide by interconnecting distant land areas and by transferring significant amounts of nutrients from the sea to land, where they deposit large amounts of nutrient near seabird colonies^13–17^. Seabird colony effects on terrestrial ecosystems are especially important in polar regions, where vegetation is generally dispersal- and nutrient-limited, and areas below seabird colonies thus support unique Arctic habitats and biodiversity^18,19^. Seabird colonies in the High Arctic include nests within scree slopes (dominated by little auk) and nests on steep cliffs (dominated by kittywakes and Brünich gillemots). A better understanding of the role and impact of sea-to-land transport of nutrients on terrestrial biodiversity and functioning represents an important first step toward better understanding of the consequences of seabird declines on terrestrial Arctic biodiversity and ecosystems.

High Arctic land areas are typically relatively isolated, often species-poor, and support unsaturated floras and faunas. Trait-based approaches present valuable opportunities for generalization and enhanced insights in these systems although questions still remain about the relevance of traits for vegetation changes in the Arctic^20^. Focusing on functional traits rather than taxonomic composition enables comparisons among plant communities with different taxonomic compositions within and across sites and regions^21,22^. At fine spatial and temporal scales, and of particular relevance in relatively species-poor High Arctic vegetation, intraspecific trait variation can inform on individual and population-level responses to global change, including response to shifting selection pressures^5,23,24^. Trait-based approaches further allow insight into the processes governing both community assembly (via “response traits”) and consequences of vegetation changes ecosystem functioning (“effect traits”)^25–27^. At landscape scales, vegetation and trait data can be combined with multispectral imagery to upscale information on plant functioning, chemistry, and water relations^28,29^. Integrating traits with data from various biological levels, including plant physiology, vegetation functioning, ecosystem dynamics, and remote sensing, can thus facilitate comprehensive assessments and enhance our understanding of how arctic biodiversity and ecosystems respond to global change at various scales levels of organization, from intraspecific to ecosystem and from plot-scale to landscapes^30,31^.

**Fig. 1:**
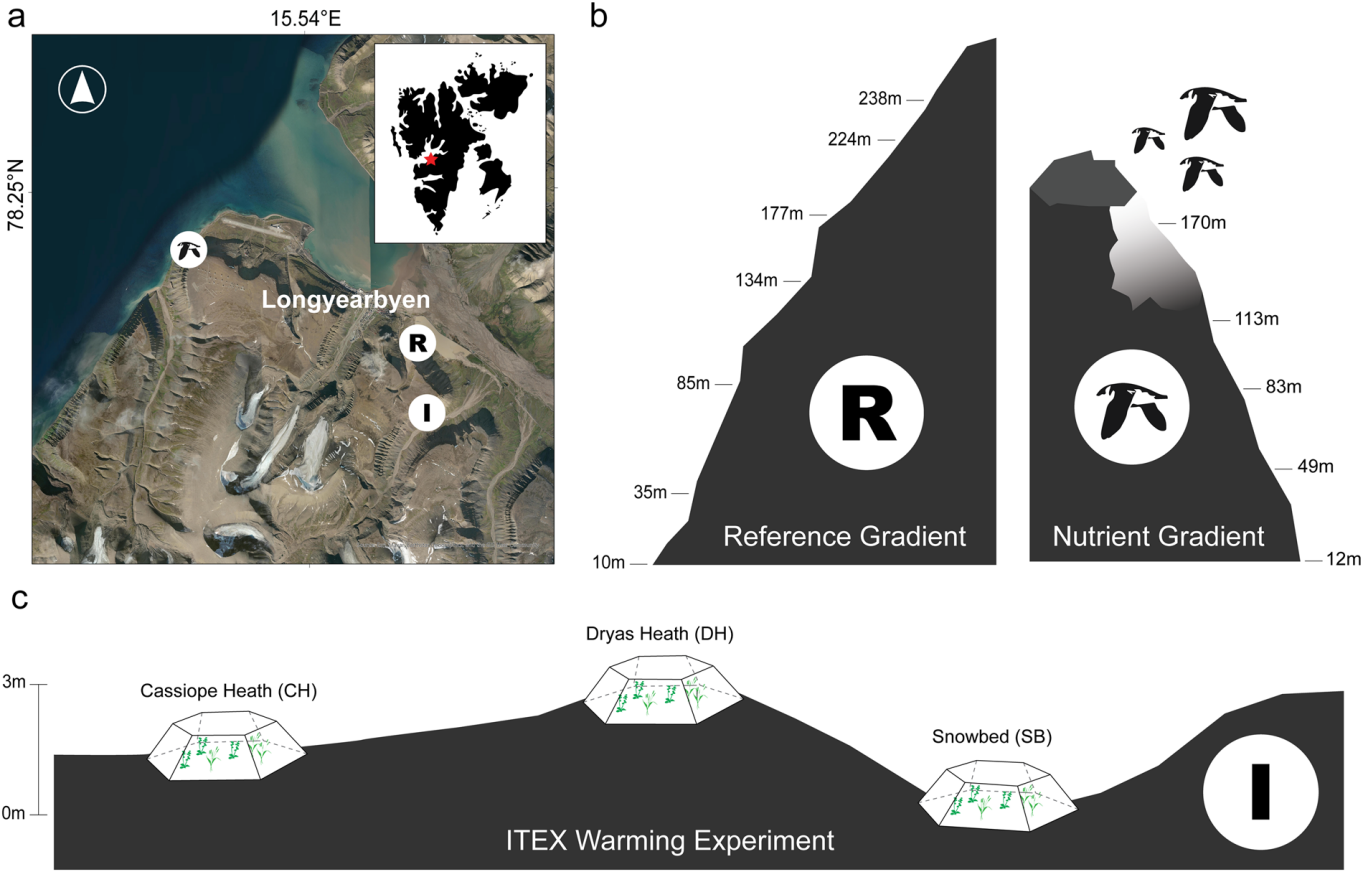
Experimental site and gradients for the traits and associated data sampling in High Arctic Svalbard. (**a**) Inset map and aerial photo showing the location of the study area on Svalbard and the location of the seabird nutrient input gradient (bird icon), reference gradient (R), and ITEX warming experiment (I) in relation to Longyearbyen. (**b**) Schematic illustration of the elevational distribution of sites (marked by their elevation) and nutrient influence (lighter area below the little auk colony) within the reference (R) and nutrient input (bird icon) gradient. (**c**) Schematic illustration of the relative topographic positions of the *Dryas* heath (DH), *Cassiope* heath (CH), and snowbed (SB) habitats, each represented by one Open Top Chamber, along a snowmelt gradient within the ITEX site (I), Note that the full ITEX site design includes five OTCs and five control plots (not shown) within each habitat.

In this study, we report on an integrated dataset combining plant functional traits with plot-scale vegetation, ecosystem, and climate data and landscape-scale multispectral imagery to assess the role of climate warming and nutrient inputs from marine sources vis seabirds on biodiversity, functional traits, ecosystem processes, and landscape patterns in High Arctic vegetation near Longyearbyen, Svalbard (Fig. 1). First, we sampled an International Tundra Experiment (ITEX, https://www.gvsu.edu/itex/) warming experiment established in 2001 spanning three different habitats along a snowmelt gradient, from dry and early melt-out *Dryas* heath via mesic *Cassiope* to moist and late melt-out snowbeds, to assess effects of climate warming on the biodiversity and functioning of High Arctic vegetation. Second, in 2018, we established two elevational gradients (from sea level to approximately 200 m a.s.l.); comparing a gradient below a seabird colony (dominated by little auks), where birds deposit nutrients from the sea, to a reference gradient with no such impact. In the ITEX experiment, vegetation community composition and climate data have been recorded three times since 2003. In a 2018 field campaign, we measured a range of functional trait-related data in both study systems, including vegetation structure, vascular plant and bryophyte functional traits, ecosystem CO_2_ and water fluxes, remote sensing, spectral reflectance, and associated microclimate data. In 2018, we also recorded species composition at the seabird colony nutrient input gradient and the reference gradient. While some of these data have been used in previous publications^5,28^, here we present and integrate all the available data from these campaigns to safeguard the data for the future, expand trait data coverage, make data available to others, and allow future exploration into biodiversity assembly, ecosystem functioning, and global change impacts in the High Arctic. Such knowledge is crucial for effective conservation and management in vulnerable Arctic and Alpine biomes.

The dataset consists of 16,160 unique trait measurements across 34 vascular plant taxa covering 52.7% of the species in the local plant communities, along with 1,048 bryophyte trait measurements from 10 abundant bryophytes (Table 1). This extends existing vascular plant trait data from the regional flora by nine taxa for which no previous trait data exists in databases or in the published literature and increases the number of unique trait measurements from this regional flora by 33%, relative to the public TRY database^32^. These data allow exploration of intraspecific trait variation in response to experimental treatments and environmental gradients, see for example^5^, and offer vegetation, ecosystem flux, reflectance, remote sensing, and microclimate data from the same sites and plots (Table 1), thereby offering opportunities for a comprehensive exploration of linkages to environmental drivers and feedback to climate. Our data were collected as part of the Plant Functional Traits Courses (PFTC4), a program for international students specializing in trait-based theory and methods (https://plantfunctionaltraitscourses.w.uib.no/), see also^33,34^. The data aligns with information from similar courses and field campaigns conducted in China^35^, Peru^36^, and Western Norway, paving the way for future comparative studies.

**Table 1:** Description and location of the datasets plant functional traits and associated data from an ITEX warming experiment and two elevation gradients, with and without marine nutrient input from nesting seabirds, near Longyearbyen, Svalbard.

Dataset	Response variable	Number of data points in ITEX^a^, gradients^b^	Number of taxa in ITEX^a^, gradients^b^	Temporal range in ITEX^a^, gradients^b^	Citation information for raw data, clean data, and code
i	Plant community composition	1,273^a^ 689^b^	26 vascular plants, 1 fungus, 8 lichens, 22 bryophytes^a^ 50 vascular plants^b^	2003, 2009, 2015^a^ 2018^b^	Raw data^66^, clean data^66^, code^67^
ii	Vegetation structure and height	61^a^ 756^b^		2003; 2009, 2015^a^ 2018^b^	Raw data^66^, clean data^66^, code^67^
iii	Vascular plant and bryophyte traits	5,339^a^ 11,345^b^ (10,297 vascular plants; 1048 bryophytes)	19 vascular plants^a^ 31 vascular plants, 19 bryophytes^b^	2018^a,b^	Raw data^66^, clean data^66^, code^67^
vi	Soil carbon and nitrogen	70^b^		2022^b^	Raw data^66^, clean data^66^, code^67^
v	Ecosystem CO_2_ fluxes	raw flux measurements 129^a^ 59^b^		2018^a,b^	Raw data^66^, clean data^66^ code^5^
vi	Remote sensing	7 sites, 28,500 (x5) individual multispectral images; 340 leaf spectroscopy readings, 117 ground-truthing points^a,b^	18 species of moss, graminoid, and dwarf shrub^a,b^	2018^a,b^	Clean data^66^
vii	Climate data	station: 815,339^a^ loggers: 937,388^a^ 162^b^		station: 2015–2018^a^ loggers: 2004–2005; and 2015–2018^a^ 2018^b^	Raw data^66^, clean data^66^, code^67^

This table summarizes information on dataset number, response variable(s), number of observations, taxa, the data’s temporal range, location of the primary data, the final published data, and the code for extracting and cleaning data from the primary data. The superscript letters refer to ^a^ITEX warming experiment, ^b^Gradients, Note: The ITEX climate data consists of two data tables; one for the climate station, one for climate logger data.

## Methods

### Data management and workflows

**Fig. 2:**
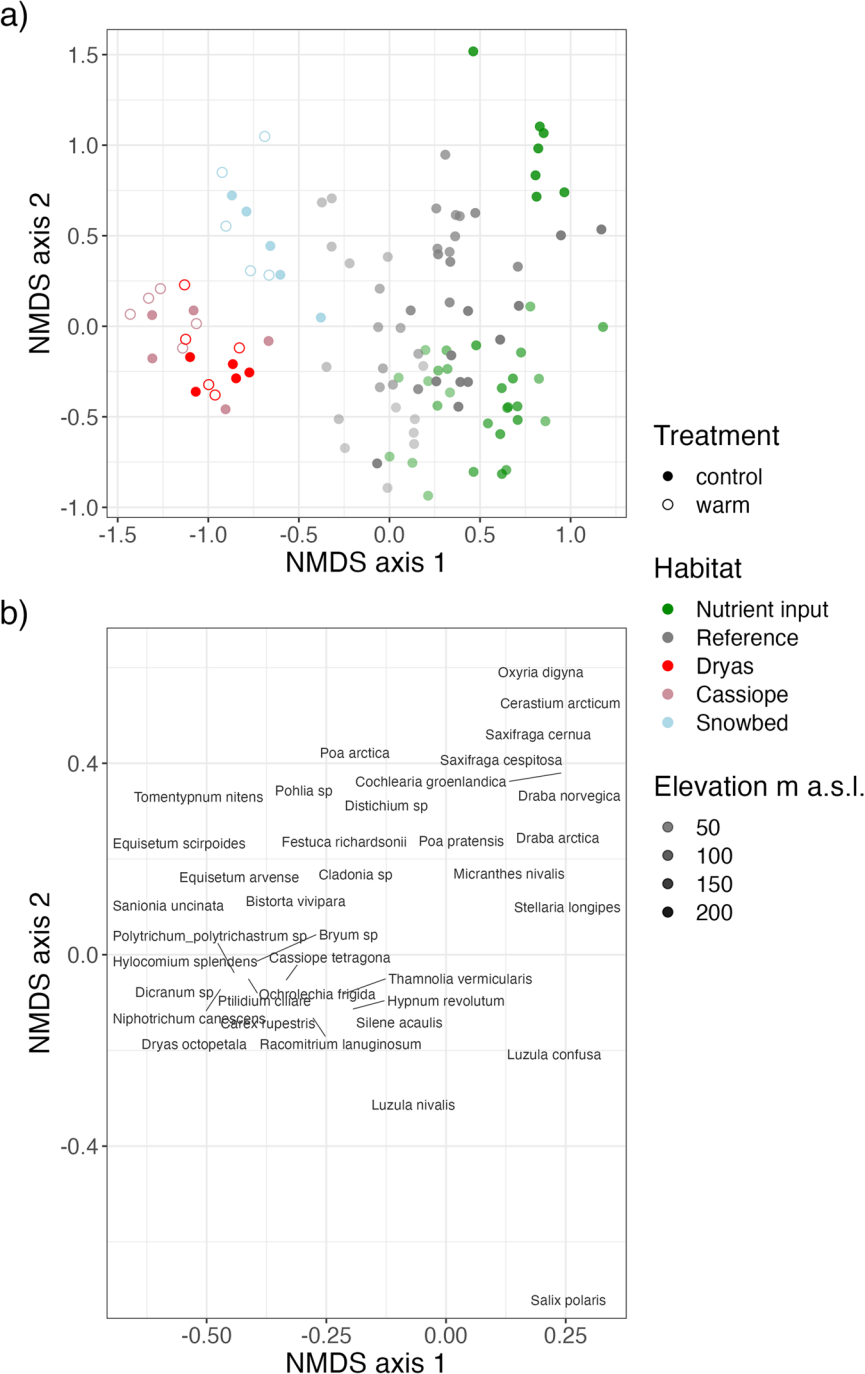
Non-metric Multidimensional Scaling (NMDS) ordination depicting variation in taxonomic composition of vascular and nonvascular plants in the 96 vegetation plots from the Endalen ITEX site (three habitats; *Dryas* heath, *Cassiope* heath, snowbed) and two elevation gradients (seabird colony nutrient input, reference). (**a**) Plot scores on NMDS axes 1 and 2, based on 74 taxa (see^67^ for details). Shapes and colors indicate habitats and experimental treatment within the two study systems (*Dryas* heath (red), *Cassiope* heath (pink), snowbed (blue)) from the ITEX warming experiment (filled: control, open: warmed), and the seabird nutrient input (green) and reference (gray) gradients. Opacity indicates elevation of the sites, with darker color corresponding to higher elevation. (**b**) Species scores of selected taxa on NMDS axes 1 and 2 (see^67^ for code to generate the NMDS scores for the full list of taxa).

We adopt best-practice approaches for open and reproducible research planning, execution, reporting, and management throughout the project (e.g.^37–40^) Specifically, we use community-approved standards for experimental design and data collection. We clean and manage the data using a fully scripted and reproducible data workflow, with data and code deposited at open repositories (see Fig. 2 in^41^ for a schematic representation of our approach to data management). The paper reports on data available in 10 main data tables, linked by keys related to time, sampling locations, and species (Fig. 2).

### Research site selection and basic site information

Our study took place in High Arctic vegetation near Longyearbyen, Svalbard (Fig. 1). We sampled a warming experiment using Open Top Chambers (OTC) in three distinct habitats along a snowmelt gradient, see^5^ and two elevational gradients, one located below a bird-cliff with nutrient input from nesting seabirds and one without the influence of sea birds. The study area is characterized by a dry Arctic climate with a mean annual temperature of −2.6 °C and annual precipitation of 190 mm^28^. The prevailing wind direction in the area is from the east, and the soils are typical cryosols with a thin organic layer on top of inorganic sediments^42^.

### ITEX warming experiment

The ITEX warming experiment is situated on the south–southeast facing hillside of Endalen (78.18°N, 15.75°E), four kilometers east of Longyearbyen, Svalbard, at 80 m a.s.l. (Fig. 1)^5^. This experiment is part of the International Tundra Experiment (ITEX), a research network established in 1990 to study the long–term responses of tundra plants and vegetation to climate warming^43,44^. The experiment was established in 2001 in three characteristic High Arctic habitats differing in the timing of snowmelt and hence the duration of the growing season (see^5^ for further description). The relatively dry *Dryas* heath (DH) is found in areas with thin snow cover (ca. 10 cm) and early snowmelt. It is dominated by *Dryas octopetala* with abundant *Carex rupestris, B. vivipara*, and *Salix polaris* as common vascular plant species. The mesic *Cassiope* heath (CH) is found in areas with medium snow depth and snowmelt dates. It is dominated by *Cassiope tetragona* with abundant *S. polaris* and *B. vivipara* as other common vascular plants. The moist snowbed (SB) habitat is found in areas with deep snow (over 100 cm) and late snowmelt. It supports more herbaceous vegetation, co-dominated by *Salix polaris, Bistorta vivipara, Poa arctica*, and *Festucarichardsonii*. Ten 75 × 75 cm plots were established within each habitat, half of which were randomly assigned to a warming treatment using Open Top Chambers (OTC) in 2002. The other half served as controls. The OTCs have a base diameter of 1.5 meters and a height of 40 cm. See^5^ for further information.

### Elevation gradients with and without marine-derived nutrient input by seabirds

In 2018, we established two elevational gradients to study the effects of marine-derived nutrient input from seabirds on High Arctic vegetation and ecosystem functioning (Fig. 1). One gradient is near Bjørndalen (78.24°N, 15.35°E), home to a seabird nesting colony predominantly occupied by the little auk (*Alle alle*). Nestled among rocky outcrops and talus slopes beneath a steep cliff, these birds deposit nutrients as guano on the slope below. Nutrient deposition should generally be highest near the nesting seabirds and decrease with increasing distance downslope. However, small-scale topography strongly influences distribution of nutrients, which are higher in concave areas and small depressions in the slope^28^. We hereafter refer to this as the ‘nutrient input gradient’. The second gradient, referred to as the ‘reference gradient’, Lindholmhøgda (78.20°N, 15.72°E), is free from seabird influence. Both elevational gradients are situated on mountains of comparable elevation and slope, share similar bedrock^45^, and are primary grazing grounds for the Svalbard reindeer (*Rangifer tarandus platyrhynchus*). The slope under the seabird colony at Bjørndalen faces northwest, beginning at the seashore. The reference gradient at Lindholmhøgda, located about one kilometer inland, faces northeast (Fig. 1).

At the nutrient input gradient, we established five study sites between 12 and 170 m a.s.l. Because the seabirds nest at the top of the slope, just below the cliffs, this is both a gradient in elevation and in marine-derived nutrient from birds, as the nutrient input from the birds increases with elevation. The highest-elevation site at this nutrient input gradient was chosen as close to the bird nests as possible while avoiding disturbing the birds, and the sites along the gradient were chosen to be equally spaced in elevation while avoiding dangerously steep terrain and convex depressions in the slope where water and nutrients accumulate^28^. At the reference gradient we established seven sites at roughly equally spaced elevations from 10 to 238 m a.s.l., avoiding exposed ridges and snowbeds. Given the absence of nesting seabirds nearby, we assumed the import of marine-derived nutrients to be low and consistent across the entire elevational gradient. These sites thus form a gradient based solely on elevation.

At each site per gradient, we set up seven vegetation plots measuring 75 by 75 cm, except for the highest site at the reference gradient, where only four plots were established due to limited vegetation coverage (n = 35 plots at the nutrient input gradient, n = 46 at the reference gradient). Plots were placed 5 meters apart on mesic High Arctic heath/dwarf-shrub dominated plant communities, again avoiding placing plots in obvious depressions, snowbeds or exposed ridges, as described for the site selection above.

These site and plot selection criteria helped minimize heterogeneity within and between gradients due to factors other than elevation and bird influence, which was especially important given the different topography at the two gradients, and the importance of topography for environmental variation and vegetation patterns within gradients^28^. We marked the plots using differential GPS and determined their aspect and slope using a digital model of the site^28^.

### Other study systems

Together with the three focal sites described above, the remote sensing team obtained Unmanned Aerial Vehicle (UAV, drone) imagery (see the descriptions of dataset iv and^28^ for details on these methods and data) from four other study systems. These are:

#### Flux tower

This site consists of a continuous permafrost polygonal tundra lowland on a river terrace on the flat part of a large alluvial fan in Adventdalen around an eddy covariance (EC) tower established in 2011 to conduct flux measurements of CO_2_ and CH_4_. Vegetation includes *Salix polaris* in drier areas and *Eriophorum scheuchzeri* and *Carex subspathacea* in wetter locations. Shrubs dominate the drier polygons and moss dominates the depressions^46^.

#### Snow fences

This site consists of an area underlain by permafrost in Adventdalen (origin 78.174387 N, 16.05769E), dominated by the dwarf shrubs *Dryas octopetala* on ridges and *Cassiope tetragona* in concavities, with *Salix polaris* throughout the site. At this site, snow regime was manipulated using snow fences^47^.

#### Valley opposite the ITEX site

We flew over the valley opposite the ITEX site. It consists of a north–northeast–facing hillside in Endalen (origin 78.18 °N, 15.76 °E), of similar topography to the ITEX site. The flight was conducted to provide a comparable site to the ITEX site but with a different aspect.

#### Alluvial fan

We conducted a drone flight over a well-defined alluvial fan with patterned ground in Adventdalen (origin 78.17 °N, 16.04 °E), not far from the Snow Fences site. Vegetation over the site was very sparse and the site was chosen to capture the geomorphological features of the fan.

### Species identification, taxonomy, and flora

All vegetation and functional trait data were based on plants sampled in the field. We collected plants or vouchers for verification, identified them using the floras available at the time of collection, and checked the final data against the Svalbardflora (https://www.svalbardflora.no/). Specimens with identification challenges, such as non-flowering *Draba* and grasses, some *Poa* spp. individuals, were assigned a descriptive name and stored as vouchers. Taxon names were standardized using the TNRS R package^48^ based on the Taxonomic Name Resolution Service^49^, Tropicos^50^, The Plant List^51^ and USDA^52^ databases. We identified bryophyte species following Swedish Nationalnyckeln for mosses^53^. All sampled species were identified as native to Svalbard.

### Dataset (i): Species community composition sampling

#### ITEX warming experiment

All vascular plant species, bryophytes, and lichens were recorded in peak growing season in each plot in 2003, 2009, and 2015 using the point intercept method as outlined in the ITEX manual^54^. We used a 75 × 75 cm frame with 100 evenly distributed points and recorded all hits within the canopy until the pin reached the cryptogam layer (composed of bryophytes and lichens), bare ground, or litter. The amount of dead plant tissue on the ground (litter), un-vegetated soil surface (bare ground), and rock was also recorded. The dataset also contains information on plant functional groups to which each species belongs (woody, graminoid, forb, bryophyte, lichen). In the winter of 2008–2009, ice formation at ground level resulted in the death of many *Cassiope* shrubs in two control plots and two OTC plots in the *Cassiope* heath, which we noted in the dataset.

#### Elevational gradients

We recorded vascular plant species composition in all plots along both elevational gradients in July 2018. In each plot, the percentage cover of all vascular plant species was visually estimated to the nearest 1%, where total coverage could exceed 100 due to vegetation layering. In addition, we recorded if species were fertile (i.e., presence of buds, flowers, and seeds). The team verified the taxonomy with available literature and databases and consulted experts as needed (see above). We also surveyed the bryophytes, using a simplified methodology due to time limitations and logistic constraints. Specifically, at three of the plots at each elevation, the three most abundant bryophyte taxa were identified. Note that these are presence-only data with no information on bryophyte abundance, and that the bryophyte community data are available via the trait dataset (see below).

### Dataset (ii): Vegetation height and structure

#### ITEX warming experiment

Vegetation height was measured in 2009 and 2015 using two different methods. In 2009 the height of the highest individuals in the center of each of four subplots in a 75 × 75 cm plot was measured. In 2015, the height was measured at 100 regularly spaced points in each plot and then averaged per plot.

#### Elevation gradients

Average and maximum vegetation height was calculated based on measurements at four randomly selected points within each plot. We also visually estimated each plot’s total cover of vascular plants, bryophytes, biocrust, lichens, litter, rocks, and bare ground.

### Dataset (iii): Vascular plant and bryophyte functional trait sampling and lab analyses

#### Plot-level vascular plant sampling for leaf trait analyses

We collected whole plants for leaf trait analysis in the ITEX warming experiment and the elevational gradients in July 2018. Three whole individuals or ramets of plants, including roots, were collected per species and plot for all species recorded with more than 1% cover in that plot. The plants were collected outside the plot but within the close surroundings (inside the OTC, for warmed plots) to not destructively sample from within the plots. To avoid repeated sampling from a single clone, we selected individuals that were visibly separated from other ramets of that species.

#### Plot-level bryophyte sampling for trait analysis

We sampled bryophyte tussocks for functional trait analysis at both elevational gradients in July 2018. Three tussocks of the three most dominant bryophyte species were sampled at three plots at each elevation site for a maximum of nine samples per site. The bryophytes were collected near but not in the plots to avoid destructive sampling within plots. The following taxa were collected: *Aulacomnium turgidum*, *Dicranum* sp., *Hylocomium splendens*, *Racomitrium canescens*, *Racomitrium* sp., *Polytrichum piliferum*, *Polytrichum* sp., *Sanionia* sp., *Syntrichia ruralis*, and *Tomentypnum nitens*.

#### Processing and storage

The samples were typically processed within one day after field collection, although some specimens were stored for up to 4 days. Collected plants were stored under cool (ca. 6 °C) moist conditions. Prior to processing, we conducted plant identification checks (see above). In the case of vascular plants, we sampled up to three healthy, fully expanded leaves from each individual. If the leaves were very small, we collected several leaves  to reach a combined area of ca. 3 cm^2^. The leaves were cut off as close to the stem as possible, including the blade, petiole, and stipules, as present. For *Equisetum*, where stems are the main photosynthetic structure, we sampled an 8 cm long section of the stem on which measurements were made (i.e., all photosynthetic tissue, including stem, branches, and microphylls).

For bryophytes, we collected at least five living (i.e., green) shoots (considering approximate biomass needed for chemical analysis), including any non-green lower parts of those shoots, from each tussock. We carefully cleaned these shoots from soil and debris using tweezers under a stereo microscope. Subsequent processing was conducted within 24 hours (see below).

#### Functional trait measurements

For *vascular plants*, we measured 14 leaf functional traits reflecting the (i) size, (ii) leaf economic trade-off in the acquisition and utilization of resources (e.g., carbon, water) that govern the potential physiological growth rates and environmental tolerance of plants, and (iii) plant nutrient status, following the standardized protocols described by Pérez-Harguindeguy *et al*.^55^: plant height (cm), leaf wet mass (g), dry mass (g), leaf area (cm^2^), leaf thickness (mm), leaf dry matter content (LDMC, g/g), specific leaf area (SLA, cm^2^/g), carbon (C, %), nitrogen (N, %), phosphorus (P, %), carbon to nitrogen ratio (C:N), nitrogen phosphorus ratio (N:P), carbon isotope ratio (δ^13^C, ‰), and nitrgen isotope ratio (δ^15^N, ‰).

For *bryophytes*, we selected easy-to-measure soft traits similar to those selected for vascular plants, related to size, trait trade-offs related to leaf economics, and nutrient status, following protocols described in^56^. Specifically, we measured wet mass (g), dry mass (g), shoot length (length of total and green living part; cm), shoot ratio, specific shoot length (SSL cm g^−1^, as described in^57^, water holding capacity (WHC, g g^−1^), carbon (C, %), nitrogen (N, %), phosphorus (P, %), carbon to nitrogen ratio (C:N), nitrogen to phosphorus ratio (N:P), carbon isotope ratio (δ^13^C, ‰), and nitrogen isotope ratio (δ^15^N, ‰). Note that because of the large morphological differences between bryophytes and vascular plants, the selected traits of the two primary producer groups may not be directly comparable.

Initial leaf processing and size and leaf economic traits were measured at the University Centre in Svalbard, Longyearbyen, Svalbard, and nutrient traits were measured at the University of Arizona, Tucson, Arizona, in the following steps:

- *Vascular plant height*. Before sampling the plants in the field, we measured standing height (measured in cm) for each individual as the distance from the ground surface to the highest tip of a photosynthetic leaf, excluding florescences but including stem leaves when relevant.
- *Vascular plant leaf wet (fresh) mass*. For vascular plants, each leaf (including blade, petiole, and stipules when present) was gently blotted with paper towels to remove excess water and any debris before it was weighted to the nearest 0.001 g using a Mettler AE200, Mettler TOLEDO, or AG204 DeltaRange (0.1 mg precision).
- *Vascular plant leaf area*. We flattened leaves to maximize their area, and scanned them using a Canon LiDE 220 flatbed scanner at 300dpi. Leaves that naturally grow folded, such as those of *Festuca* species, were scanned as such, and the area was then doubled during data processing. Any dark edges on the scans were automatically removed during data processing. Leaf area was calculated using ImageJ^58^ and the LeafArea package^59^.
- *Vascular plant leaf thickness*. Leaf thickness was measured at three locations on each leaf blade with a digital caliper (Mitutoyo 293–348), and the average was calculated for further analysis. When possible, the three measurements were taken on the middle vein of the leaf and the lamina with and without veins. The petiole or stipule thickness was not measured.
- *Vascular plant leaf dry mass*. Leaves (including blade, petiole, and stipules when present) were then dried for at least 72 hours at 60 °C before dry mass was measured to the nearest 0.0001 g.
- We calculated *vascular plant specific leaf area* (SLA) by dividing leaf area by dry mass and *Leaf dry matter content* (LDMC) as the ratio of dry to wet mass.
- *Bryophyte shoot length*. The stretched length of three shoots (both the length of the total shoot and the length of the green living part) of each bryophyte sample was measured. In cases where the shoots had multiple tips, only the longest (main) shoot was measured.
- *Bryophyte wet mass*. First, the bryophyte shoots were soaked in demineralized water for 30 minutes. Subsequently, shoots were kept in sealed Petri-dishes lined with moist tissue paper overnight to ensure full water saturation. Then, the shoots were blotted dry with tissue paper and weighed for wet mass (AG204 DeltaRange, Mettler Toledo).
- *Bryophyte dry mass*. The samples were dried to a constant mass at 60 °C for 72 h and weighed.
- *Bryophyte water holding capacity (WHC)*. WHC was expressed as (wet mass – dry mass)/dry mass (g g^−1^).
- *Bryophyte specific shoot length (SSL)*. SSL was calculated by dividing the total shoot length by its dry mass (cm g^−1^).
- *Vascular plant leaf and bryophyte shoot stoichiometry and isotopes*. A subset of leaves (ITEX: n = 2,405; reference: n = 1,596; nutrient input = 1,384) and bryophyte shoots (n = 304) were sent to the University of Arizona for leaf stoichiometry and isotope assays (P, N, C, δ^15^N, and δ^13^C). The samples were stored in a drying oven at 65 °C before shipping and processing. Each leaf (including blade, petiole, and stipules when present) or bryophyte shoot sample was ground into a fine homogenous powder for measurements.
- We determined the total *phosphorus* concentration using persulfate oxidation and the acid molybdate technique (APHA 1992), followed by colorimetric measurement of the phosphorus concentration with a spectrophotometer (TermoScientifc Genesys20, USA).
- *Nitrogen, carbon, stable nitrogen* (δ^15^N), and *carbon* (δ^13^C) isotopes were measured in the Department of Geosciences Environmental Isotope Laboratory at the University of Arizona using flash combustion analysis of organic matter via a continuous-flow gas-ratio mass spectrometer (Finnigan Delta PlusXL) coupled to an elemental analyzer (Costech). The process involved combusting samples of 1.0 ± 0.2 mg in the elemental analyzer. Standardization relied on acetanilide for elemental concentration, NBS-22 and USGS-24 for δ^13^C, and IAEA-N-1 and IAEA-N-2 for δ^15^N. Precision is at least ± 0.2 for δ^15^N (1 s), based on repeated internal standards.
- Finally, we calculated ratios between *C:N* and *N:P*.

### Dataset (iv): Soil carbon and nitrogen sampling

Samples of the top 5 cm of soil, including both the organic and mineral soil layer, were taken at the end of July and August 2022 in both elevational gradients. At each elevational site, we took three random soil samples, except for the middle site at the nutrient input gradient, where we only took 2 samples due to a sampling error (nutrient input: n = 14; reference: n = 21). For each sample, we used a soil corer with a diameter of 5.7 cm. Litter and above-ground vegetation, including vascular plants and live parts of cryptogams (bryophytes, lichens, soil crust, if present), were removed from the top of each sample before the soil cores were cut at 5 cm below the soil surface. The samples were pre-dried in the lab at 30 °C for at least one week, then properly dried for two days at 60 °C. Stones and roots were removed using a 2 mm sieve. The resulting soil samples thus did not contain live above- or below-ground vascular or cryptogam plant material but included any dead parts of the cryptogamic community along with litter embedded in the soil profile. Carbon and nitrogen content were analyzed from well-mixed subsamples using dry combustion^60^.

### Dataset (v): Ecosystem CO_2_ fluxes

#### Plot-level flux measurements

In July 2018, ecosystem CO_2_ fluxes were measured in all plots at the ITEX warming experiment. We also measured ecosystem CO_2_ fluxes at the highest elevation (site 5, 170 m a.s.l.) at the nutrient input gradient and all sites except the lowest along the reference gradient (site 2–7). Due to bad weather conditions, we could not measure fluxes in all the plots and sites along the two elevational gradients.

To estimate Net Ecosystem Exchange (NEE), ecosystem respiration (R_eco_), Gross Primary Production (GPP), and soil respiration (Rs), following^61^, we employed a static chamber method to measure CO_2_ fluxes. The chamber, constructed from plexiglass with dimensions of 25 × 25 × 40 cm, featured two fans for air circulation and was connected to an infrared gas analyzer (Li-840, LI-COR Biosciences, Lincoln, NE, USA) to measure CO_2_ fluxes.

For each plot, CO_2_ fluxes were measured twice: once under light conditions, and once under dark conditions. Light measurements, taken during cloud-free conditions, captured photosynthetic CO_2_ uptake and respiratory CO_2_ release from the ecosystem. For dark measurements, an opaque hood was employed to block out sunlight^62^, thereby ceasing photosynthesis and enabling the measurement of respiratory CO_2_ release from the ecosystem, encompassing both plant and soil respiration.

For each measurement, continuous measurements of ambient CO_2_ and H_2_O were taken for 30 seconds before placing the chamber over the plot and measurements then continued for approximately 90 seconds within the chamber. The fans ensured efficient mixing of ambient air and air mixing inside the chamber. Previous studies have demonstrated that after 90 seconds, changing concentrations of CO_2_ and H_2_O in the closed system begin to impact stomatal conductance^63,64^. This duration also mitigates the influence of increasing temperature on the plants within the chamber. The chamber’s closure was achieved by sealing it with a long canvas skirt along the base, weighed down with a heavy chain. To equilibrate air conditions inside the chamber with the ambient air, the chamber was aired for 1 minute between each measurement.

#### Soil respiration measurements

Soil respiration was measured in all transect sites except for the lowest elevation at both gradients. In each plot, we inserted a PVC collar with a diameter of 10 cm to function as the chamber space for soil respiration measurements. We measured the height of each collar at four points to calculate the volume of the collar for flux calculations.

#### Environmental measurements

We measured environmental data in each plot during the ecosystem CO_2_ fluxes measurements or right before/after. Photosynthetic active radiation (PAR; µmol photons m-2 s-1) was recorded approximately every 30 seconds during the 90–120 second measuring interval using a quantum sensor (Li-190, LI-COR Biosciences, Lincoln, NE, USA). Soil moisture (% volume) was measured using a ML3 ThetaProbe Soil Moisture Sensor from Delta-T Devices at four points evenly distributed within each plot after each measurement in dark conditions of the chamber and twice for the soil respiration collars. Soil temperature (°C) was measured using a digital thermometer with an accuracy of ±0.1 °C at two locations within each plot and each soil respiration collar during all flux measurements. Finally, canopy temperature (°C) with an accuracy of ±0.1 °C at vegetation level, was measured with an IR-thermometer with a laser pointer. For each plot, three measurements were evenly distributed across the plot after each flux measurement.

#### Calculations

NEE was calculated from the light measurements, R_eco_ from the dark measurements, and GPP from both measurements as follows (Note that calculations were done for the ITEX warming experiment data only). All raw flux data were visually evaluated for quality, and only measurements that showed a consistent linear relationship between CO_2_ and time for at least 60 s were used for calculations. NEE was calculated from the temporal change of CO_2_ concentration within the closed chamber during light measurements according to the following formula:

$$NEE=\frac{\delta C{O}_{2}}{\delta t}\times \frac{P\times V}{R\times A\times (T+273.15)}$$

where δCO_2_/δt is the slope of the CO_2_ concentration against time (µmol mol^-1^ s^-1^), P is the atmospheric pressure (kPa), R is the gas constant (8.314 kPa m3 K-1 mol-1), T is the air temperature inside the chamber (°C), V is the chamber volume (m^3^), A is the surface area (m^2^), and 273.15 converts temperature from degrees Celsius to Kelvin. R_eco_ were calculated in the same way from dark measurements.

We define NEE such that negative values reflect CO_2_ uptake in the ecosystem, and positive values reflect CO_2_ release from the ecosystem to the atmosphere. GPP was calculated from light and dark measurements using this formula: GPP = NEE + R_eco_.

### Dataset (vi): Remote sensing

UAV multispectral imagery, leaf spectroscopy, and ground-truthing data were collected in the ITEX warming experiment and the two elevation gradients (nutrient input and reference) in July 2018. Further, we conducted flights and collected drone multispectral imagery in four other study systems (see above), named “Alluvial Fan”, “Flux tower”, “Snow Fences”, and “ITEX_ValleyOpposite” (see above and^28^ for description of these sites and systems).

#### UAV imagery acquisition

UAV imaging data were acquired from all sites using a 3DR Solo drone equipped with a 5-band multispectral camera and light sensor (MicaSense RedEdge-MX multispectral camera - which measures surface reflectance at five narrow bands: blue (475 nm), green (560 nm), red (668 nm), Red Edge (717 nm) and NIR (840 nm) - and the MicaSense RedEdge Downwelling Light Sensor). The drone was flown at 40–60 m above the ground (depending on the elevational gradient present at each site), resulting in imagery with a pixel resolution finer than 7 cm in all cases. Multiple overlapping flights were done to cover the seven study areas. Ground control points (GCPs) for georeferencing were taken using the Emlid Reach + differential GNSS system. The drone imagery was processed in Pix4Dmapper (v.4.3.31, Pix4D, Lausanne, Switzerland) using a workflow whereby images from all flights were processed in the same project to form a single orthomosaic per site. Each orthomosaic was georeferenced with GCPs and radiometrically calibrated using a MicaSense reflectance panel as a calibration target and the readings from the Downwelling Light Sensor onboard the drone.

#### Vegetation sampling for leaf spectroscopy and ground-truthing (turfs)

At the ITEX warming experiment and the nutrient input gradient, we collected 68 20 × 20 cm single-species turfs, representing the most common plant functional types identified across all sites (bryophytes, graminoids, and dwarf shrubs). We extracted high-accuracy GNSS coordinates from the locations of the extracted turfs. The turfs were cut to a substrate depth of approximately 5 cm, sealed inside plastic bags, and transported back to the lab for species identification (as described above) and further analysis. Turfs representing a range of tissue degradation, probably as a result of drought or frost damage^65^, were also collected from the dry and mesic heaths at the ITEX warming experiment. The extent of tissue degradation was assessed on all samples and labeled as ‘Healthy’, ‘Medium’, ‘Severe’ or ‘Dead’.

#### Field spectroscopy measurements

From each turf field, we did spectroscopy measurements in the lab (ASD Fieldspec Pro; Analytical Spectral Devices, Boulder, CO, USA), taking the reflectance measurements within 24 h of turf cutting. If multiple plant species were present across the turf, we only took measurements from areas where the main species dominated. The contact probe was pushed firmly down onto the turf, so all extraneous light was excluded from the measurement. We undertook five measurements at different locations across each turf.

#### Plant trait sampling

From each healthy turf, we cut three 5 × 5 cm vegetation samples. For each of these samples, we harvested all vegetation and substrate. Fresh and dry mass was weighed, and leaf traits measured as described above (see dataset (iii), Functional trait measurements).

#### Ground-truthing vegetation points

We further obtained additional high-accuracy GNSS coordinates for vegetation points from all three core study areas (ITEX warming experiment and the two elevation gradients).

For more details on how these data were collected and processed, see^28^.

### Dataset (vii): Climate data

#### ITEX warming experiment

We recorded air temperature, precipitation, and humidity two meters above the ground from 2015 to 2018 using a automatic weather station (HOBO H21-002, Bourne, MA, USA) placed in the Dryas heath habitat, measuring air temperature (HOBO S-THB-M008) and photosynthetic radiation (PAR) (HOBO PAR S-LIA-M003) at 2 m height above ground and soil water content (HOBO S-SMC-M005) at 5 cm depth. The data were collected at 10-minute intervals. For soil temperature measurements, we employed iButtons (iButtonLink Technologies, USA) at a depth of −5 cm and surface level in 3–4 plots per habitat and treatment (n = 20) during 2014, 2015, 2017, and 2018. These measurements were recorded at 3-hour intervals. Additionally, soil temperature and surface temperature at the same depth as the iButtons were measured in three plots per habitat and treatment (n = 18) using TinyTags from 2004 to 2005. The data for these measurements were recorded at 1-hour intervals.

#### Elevational gradients

We installed temperature loggers (ThermoChron iButtons, San Jose, CA, USA) at c. 7.5 cm below the soil surface in all plots along the elevational reference (n = 46) and nutrient input gradients (n = 35). Soil temperature was measured at 4-hour intervals throughout 19.7.2018 - 10–8.2018. Further, we recorded plot-level snapshot data on soil temperature and soil moisture in all plots along the elevation reference (n = 46) and nutrient input gradients (n = 35) on the 19. July 2018. For these measurements, we used hand-held time-domain reflectometry sensors to measure volumetric water content (VWC %) at three points up to a depth of 7.5 cm (FieldScout TDR 300; Spectrum Technologies, Plainfield, IL, USA). We used high-accuracy digital thermometers measuring soil temperature (°C) in the center of each plot up to a depth of 7.5 cm (TD 11 Thermometer; VWR International bcba; Leuven, Belgium).

### Additional data

We also measured photosynthesis-temperature response curves for *Alopecurus boreale*, *Bistorta vivipara*, *Cerastium arctica*, *Oxyria digyna*, *Ranunculus sulphureus*, and an unidentified rosette. These data will be integrated with data from other sites and published in a companion manuscript (Michaletz *et al*. in prep).

## Data Records

This paper reports on data from an ITEX warming experiment and two elevation gradients, a gradient affected by marine-derived nutrients from birds nesting at the top of the slope and a reference gradient without such influence, in High Arctic vegetation near Longyearbyen, Svalbard (Fig. 1). It contains data on plant community composition, vegetation structure, plant functional traits, soil C and N, ecosystem CO_2_ fluxes, remote sensing and environmental data collected between 2003 and 2022. Data outputs consist of eight datasets, the (i) species composition, (ii) vegetation height and structure, (iii) plant functional traits, (iv) soil carbon and nitrogen, (v) ecosystem CO_2_ fluxes, (vi) remote sensing data and (vii) climate data sampled from the ITEX warming experiment and along the gradients (Table 1). Remote sensing data exists from some additional sites (see description for “dataset vii” under Methods) and other data also exists from these sites (see additional data). The data presented here were checked and cleaned according to the procedures described under Methods and Technical validation before final data files and associated metadata were produced.

The final data files (see Table 1 for an overview) and all raw data, including leaf scans, are available at Open Science Framework (OSF)^66^. For each data type, we provide separate files for the ITEX warming experiment and the gradients (Table 1). The code necessary to access the raw data and produce these cleaned datasets, as well as the calculations and statistical tests in the Data Records section, is available in an open GitHub repository, with a versioned copy archived in Zenodo^67^. The reader is referred to the code and the detailed coding, data cleaning, and data accuracy comments and the associated raw and cleaned data and metadata tables for detailed information about the data cleaning process. The Usage Notes section in this paper summarizes the data accuracy and data cleaning procedures, including caveats regarding data quality and our advice on ‘best practice’ data usage.

### Dataset (i): Plant community composition

The plot-level plant community dataset from the ITEX warming experiment has a total of 57 taxa and 1,273 observations (taxa × plots × years) (Tables 1, 2). The overall mean species richness per plot, treatment, and year (mean ± SE) is 14.14 ± 0.33 species, including vascular plants, bryophytes and lichens. The species richness ranges from 11.4 ± 0.57 in the snowbed (SB) via 14.33 ± 0.46 in the *Cassiope* heath to 17.0 ± 0.53 in the *Dryas* heath (DH). Shannon diversity and evenness show the same pattern. For more details on diversity and community responses, see^5^.

**Table 2:** Data dictionary for vascular plant community composition (dataset i-a) from an ITEX warming experiment in Endalen, Svalbard.

Variable name	Description	Variable type	Variable range or levels	Units	How measured
Year	Year of sampling	numeric	2003–2015	yyyy	recorded
Site	Site as the habitat; DH = Dryas heath, CH = Cassiope heath, SB = snowbed	categorical	CH - SB		defined
Treatment	Warming treatment; CTL = ambient conditions, OTC = warmed by Open top chamber (OTC)	categorical	CTL - OTC		defined
PlotID	Unique ID as the combination of Site and number; SB-1	categorical	CH-1 - SB-9		defined
Taxon	Species name including genus and species	categorical	alectoria nigricans - unidentified pleurocarp moss sp		identified
Abundance	Estimated species abundance	numeric	1–191		recorded
FunctionalGroup	Plant functional group; graminoid, forb, dshrub (deciduous shrub), eshrub (evergreen shrub), moss, liverwort, lichen and fungi	categorical	dshrub - moss		recorded
Elevation_m	Elevation of site	numeric	80–80	m a.s.l.	recorded
Latitude_N	Latitude of site	numeric	78.183 - 78.183	Degree north	recorded
Longitude_E	Longitude of site	numeric	15.75 - 15.75	Degree east	recorded
Flag	Flagging problems in the data	categorical	Iced - Iced		recorded

The dataset contains 1,273 observations of the covers of 57 taxa in 30 vegetation plots sampled across three different habitats, over a period of 12 years. Variable names, description variable type, range or levels, units and short description is given for all variables.

The plot-level plant community dataset from the gradients has a total of 50 taxa and 698 observations (taxa × plots × years) (Tables 1, 3). Mean species richness (including graminoids, forbs and bryophytes) per plot is 10 ± 0.58 species for the reference gradient and 6.8 ± 0.69 for the nutrient input gradient. Shannon diversity and evenness were also slightly higher at the reference gradient.

**Table 3:** Data dictionary for vascular plant community composition (dataset i-b) from elevational gradients with and without nutrient input from a seabird colony, in Bjørndalen and on Lindholmhøgda, respectively, near Longyearbyen, Svalbard.

Variable name	Description	Variable type	Variable range or levels	Units	How measured
Year	Year of sampling	numeric	2018 - 2018	yyyy	recorded
Date	Date of sampling	date	2018-07-17 - 2018-07-21	yyyy-mm-dd	recorded
Gradient	Elevational gradient; C = reference, B = nutrient input gradient input (seabird colony)	categorical	B - C		defined
Site	Site as a number 1–7	numeric	1–7		defined
PlotID	Plot ID from A to G	categorical	A - G		defined
Taxon	Species name including genus and species	categorical	alopecurus ovatus - unknown sp		identified
Cover	Estimated species cover	numeric	0–70		recorded
Fertile	Numeric value indicating if an indiviual is fertile (1; i.e. presence of buds, flowers, and seeds) or not (0).	numeric	0–10		recorded
Weather	Weather during sampling	categorical	cloudy - windy		recorded
Elevation_m	Elevation of site	numeric	9.759 - 238.159	m a.s.l.	recorded
Longitude_E	Longitude of site	numeric	15.34 - 15.712	Degree east	recorded
Latitude_N	Latitude of site	numeric	78.207 - 78.239	Degree north	recorded

The dataset contains 698 observations of the covers of 50 taxa in 63 vegetation plots sampled at the two elevational gradients in 2018. Variable names, description variable type, range or levels, units, and short description is given for all variables.

A Non-metric Multidimensional Scaling (NMDS) ordination diagram of all vegetation plots shows gradual variation in species composition within and across our two study systems (Fig. 2a). The ITEX warming experiment is found on the left-hand side of the diagram, characterized by *Dryas octopetala*, *Equisetum* spp., *Bistorta vivipara*, and a number of bryophytes (Fig. 2b). The *Dryas* and *Cassiope* heath overlap in community composition and are located towards the lower parts of the diagram whereas the snowbed is the most distinct among the ITEX habitat types, located further up and to the right in the diagram and is thus more similar to the vegetation at the lower part of the reference gradient. Within all ITEX habitats, the warmed plots (open symbols) are generally located further to the upper left in the diagram than the respective controls (filled plots). The two elevation gradients are found at the right-hand side of the NMDS space. Within each gradient, lower-elevation plots are found near the center of the diagram and higher-elevation plots are found further to the right, so that NMDS axis 1 partly reflects a temperature gradient form warmer sites and treatments at the left-hand side to colder sites at the right. Accordingly, several species characteristic of colder habitats are found at the center to right-hand side of the plot, including *Draba* species, *Luzula* spp., and *Salix polaris*. The nutrient input gradient is generally found further to the right in the diagram relative to the reference gradient, reflecting nutrients as an additional factor towards the far-right parts of NMDS axis 1. In particular, the highest-elevation plots from the nutrient input gradient, which are most affected by deposition of marine-derived nutrients from the seabirds, are relatively distinct and form a cluster at the upper far right-hand side of the diagram, characterized by several nutrient-demanding species such as *Cochleria groenlandica*, *Oxyria digyna, Cerastium arcticum, Draba* spp., and *Saxifraga* spp. In contrast, towards the lower right-hand end of the ordination diagram are species characteristic of relatively nutrient-poor habitats, including *Salix polaris*, *Luzula confusa* and *L. nivalis*. Note that small species pool and high intraspecific trait variation of High Arctic plants in Svalbard^5^ implies that many species have wide habitat and environmental ranges, and are thus found across our study systems, treatments, and gradients.

For an overview of the clean datasets and links to the code to clean and extract these data from the raw data, see Table 1. The final clean data are provided in the “Community” folder, a species list over species and experiments is provided in the same folder, and the raw data are provided in the “RawData” folder on OSF^66^. The code to download and clean the data can be found in the GitHub repository^67^ in the file R/community_plan.R.

### Dataset (ii): Vegetation height and structure

Vegetation height and structure data from the ITEX warming experiment has a total of 60 observations (site × treatment × plot; Tables 1, 4). Vegetation height did not differ between the control and warming treatment or among habitats, except in 2009, where height was lower in the *Dryas* heath (E = −2.18 ± 0.651, t_5, 24_ = −3.35, *P* = 0.003).

**Table 4:** Data dictionary for community height (dataset ii-a) from an ITEX warming experiment in Endalen, Svalbard.

Variable name	Description	Variable type	Variable range or levels	Units	How measured
Site	Site as the habitat; DH = Dryas heath, CH = Cassiope heath and SB = snowbed	categorical	CH – SB		defined
Treatment	Warming treatment; CTL = ambient conditions, OTC = warmed by Open top chamber (OTC)	categorical	CTL – OTC		defined
PlotID	Unique ID as the combination of Site and number; SB-1	categorical	CH-1 – SB-9		defined
Year	Year of sampling	numeric	2009 – 2015	yyyy	recorded
Height	Vegetation height recording height at 100 pinpoint (2015) or highest individuals (2009)	numeric	0.423 – 7.35	cm	recorded
Method	Methods used to measure vegetation height	categorical	highest_ind – pinpoint		recorded
Flag	Flagging problems in the data	categorical	Iced – Iced		recorded

The dataset contains 60 observations of 30 vegetation plots in 2009 and 2015. Variable names, description variable type, range or levels, units and short description is given for all variables.

Vegetation height and structure data from the gradient has a total of 756 observations (gradient × site × plot × variable; Tables 1, 5). Vegetation height increased with increasing elevation at the nutrient input gradient (E = 0.01 ± 0.004, t_1,71_ = 2.56, *P* = 0.013), but not at the reference gradient. The vascular plant cover decreased with elevation (E = −0.06 ± 0.026, t_1,71_ = −2.14, *P* = 0.035), but did not differ between the two gradients. Bryophyte cover increased with elevation, but more so at the nutrient input gradient (E = 0.24 ± 0.08, t_1,71 = _3.02, *P* = 0.010). Litter cover did not vary with elevation or between the gradients.

**Table 5:** Data dictionary for community height and structure (dataset ii-b) from elevational gradients with and without nutrient input from a seabird colony, in Bjørndalen and on Lindholmhøgda, respectively, near Longyearbyen, Svalbard.

Variable name	Description	Variable type	Variable range or levels	Units	How measured
Year	Year of sampling	numeric	2018 - 2018	yyyy	recorded
Date	Date of sampling	date	2018-07-17 - 2018-07-21	yyyy-mm-dd	recorded
Gradient	Elevational gradient; C = reference, B = nutrient input gradient input (seabird colony)	categorical	B – C		defined
Site	Site as a number 1–7	numeric	1–7		defined
PlotID	Plot ID from A to G	categorical	A – G		defined
Value	Height or cover value	numeric	0 – 98	cm, percentage	recorded
Elevation_m	Elevation of site	numeric	9.759 – 238.159	m a.s.l.	recorded
Longitude_E	Longitude of site	numeric	15.34 – 15.712	Degree east	recorded
Latitude_N	Latitude of site	numeric	78.207 – 78.239	Degree north	recorded

The dataset contains 756 observations in 81 vegetation plots sampled across the two gradients in 2018. Variable names, description variable type, range or levels, units and short description is given for all variables.

For an overview of the clean datasets and links to the code to clean and extract these data from the raw data, see Table 1. The final clean data are provided in the “Community” folder, and the raw data are provided in the “RawData” folder on OSF^66^. The code to download and clean the data can be found in the GitHub repository^67^ in the file R/community_plan.R.

### Dataset (iii): Plant functional traits

In the ITEX warming experiment, we measured size, leaf economic,  and nutrient traits (plant height, wet mass, dry mass, leaf area, leaf thickness, specific leaf area [SLA], and leaf dry matter content [LDMC], Carbon [C], Nitrogen [N], Phosphorus, C:N and NP ratios, and isotope ratios [δ^13^C, δ^15^N]) for 436 leaf samples from 19 taxa across all sites and treatments, for a total of 5,339 trait observations (site × treatment × plot; Tables 1, 6). We also happened to sample three leaves of a lonely individual of *Betula nana* we encountered growing close to the site. There are similar numbers of leaves per site (DH = 1,894; CH = 1,737; SB = 1,666) and treatment (CTL = 2,691; OTC = 2,606).

**Table 6:** Data dictionary for plant functional traits (dataset iii-a) from an ITEX warming experiment in Endalen, Svalbard.

Variable name	Description	Variable type	Variable range or levels	Units	How measured
Project	Project from where data were collected; ITEX = ITEX warming experiment, Gradient = elevational gradients	categorical	ITEX – ITEX		recorded
Year	Year of sampling	numeric	2018 – 2018	yyyy	recorded
Date	Date of sampling	date	2018-07-19 – 2018-07-25	yyyy-mm-dd	recorded
Site	Site as the habitat; DH = Dryas heath, CH = Cassiope heath and SB = snowbed	categorical	CH – SB		defined
Treatment	Warming treatment; CTL = ambient conditions, OTC = warmed by Open top chamber (OTC)	categorical	CTL – OTC		defined
PlotID	Unique ID as the combination of Site and number; SB-1	categorical	8-OTC – SB-9		defined
Individual_nr	Individual number	numeric	1–5		defined
ID	Unique leaf ID consisting of 3 letters and 4 numbers	categorical	AEC8296 – CMO6669		defined
Taxon	Species name including genus and species	categorical	lopecurus ovatus – trisetum spicatum		identified
Trait	Plant functional leaf trait including plant height, wet/dry mass, leaf area, leaf thickness, specific leaf area, leaf dry matter content, carbon, nitrogen and phosphorus content, CN and NP ratio, d13C and d15N isotope ratio	categorical	C_percent – Wet_Mass_g		defined
Value	Leaf trait value	numeric	−34.173 – 401.5	cm, g, cm2, mm, cm2/g, percentage, permil	recorded
Elevation_m	Elevation of site	numeric	Inf–Inf	m a.s.l.	recorded
Latitude_N	Latitude of site	numeric	Inf–Inf	Degree north	recorded
Longitude_E	Longitude of site	numeric	Inf–Inf	Degree east	recorded
Functional_group	Plant functional group; vascular	categorical	vascular – vascular		identified

The dataset contains 5,339 observations of the covers of 19 taxa in 30 vegetation plots sampled across three habitats in 2018. Variable names, description variable type, range or levels, units and short description is given for all variables.

**Fig. 3:**
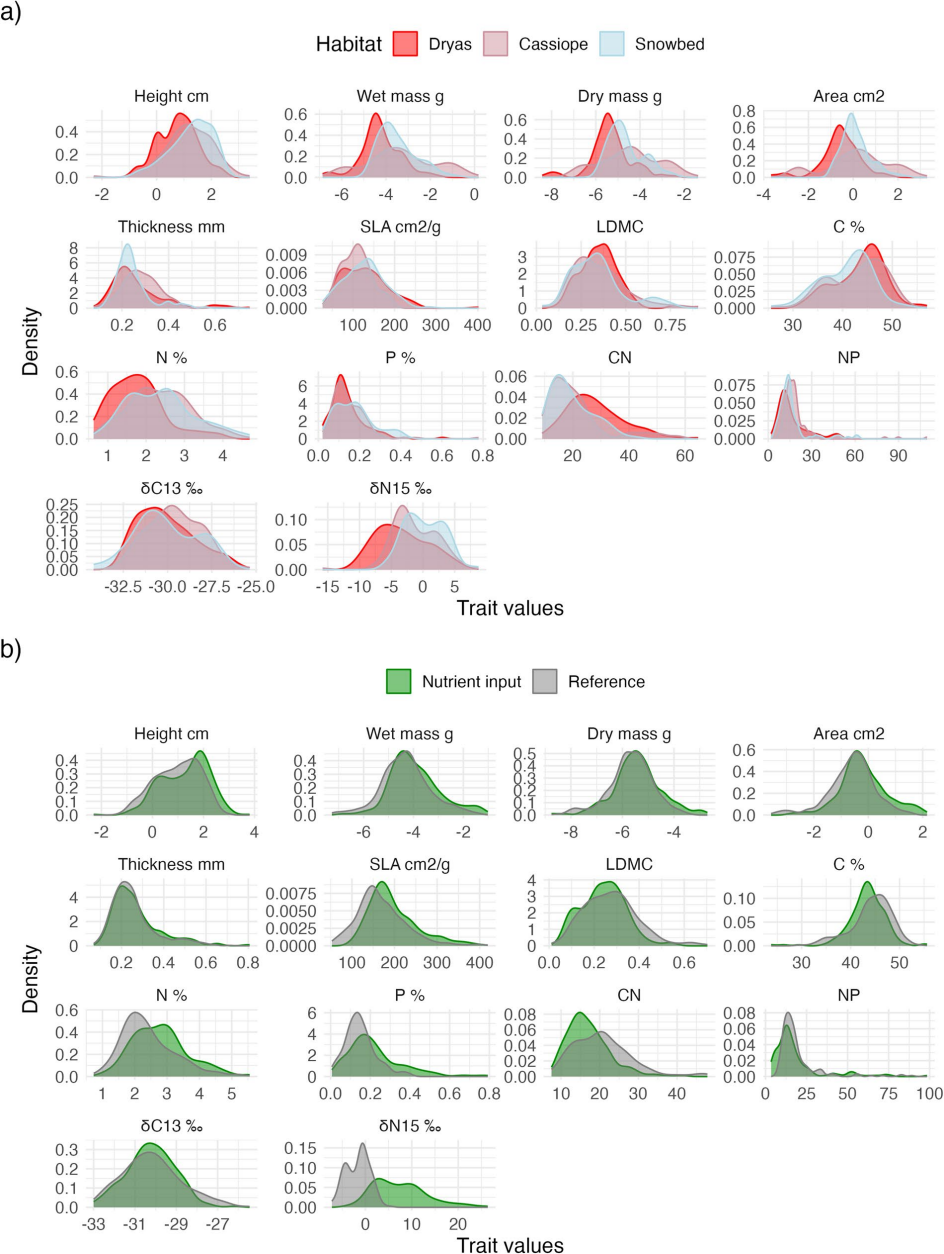
Unweighted trait distributions from (**a**) the ITEX warming experiment in Endalen and (**b**) the two elevational gradients with and without seabird colony nutrient input, in Bjørndalen and on Lindholmhøgda, respectively, near Longyearbyen, Svalbard. Distributions are given for three habitat types (*Dryas* heath, *Cassiope* heath, snowbed) within the ITEX experiment and for the two elevational gradients. The plots are based on all sampled leaves, using local trait values for each plot when available. The size traits (height, mass, length, area and thickness) are log-transformed. Note that 4 values at the elevational gradients where N:P ratio was >100 were removed before plotting the figure.

Visual inspection of the unweighted trait distributions show that “size-related traits” such as height, mass, and area tend to increase towards habitats with more snow cover (Fig. 3a). Further, leaves from snowbeds tend to have a higher carbon content and δ^15^N and lower nitrogen compared to leaves from the drier *Dryas* heath, whereas leaves from the *Cassiope* heath have intermediate values. None of the other unweighted trait distributions show clear trends. For more detailed analyses and interpretation of the trait responses, see^5^.

Along the gradients, we measured size, leaf economic, and nutrient traits (plant height, wet mass, dry mass, leaf area, leaf thickness, shoot length, shoot ratio, specific leaf area [SLA], and leaf dry matter content [LDMC], water holding capacity [WHC], specific shoot length [SSL], carbon [C], nitrogen [N], phosphorus, C:N and N:P ratios, and isotope ratios [δ^13^C, δ^15^N]) for 1,181 leaf samples from 41 taxa across all plots and both gradients, for a total of 11,345 trait observations (site × treatment × plot; Tables 1, 7). Of those 1,048 observations and 10 taxa were from bryophytes. The number of samples differed between the two gradients (reference = 7,061; nutrient input = 4,284).

**Table 7:** Data dictionary for plant functional traits (dataset iii-b) from an ITEX experiment in Endalen and two elevational gradients with and without nutrient input from a seabird colony, in Bjørndalen and on Lindholmhøgda, respectively, near Longyearbyen, Svalbard.

Variable name	Description	Variable type	Variable range or levels	Units	How measured
Project	Project from where data were collected; ITEX = ITEX warming experiment, Gradient = elevational gradients	categorical	Bryophytes – Gradient		recorded
Year	Year of sampling	numeric	2018 – 2018	yyyy	recorded
Date	Date of sampling	date	2018-07-17 – 2018-07-24	yyyy-mm-dd	recorded
Gradient	Elevational gradient; C = reference, B = nutrient input gradient (seabird colony)	categorical	B - C		defined
Site	Site as a number 1–7	categorical	1–7		defined
PlotID	Plot ID from A to G	categorical	A–G		defined
Individual_nr	Individual number	numeric	1–5		defined
ID	Unique leaf ID consisting of 3 letters and 4 numbers	categorical	AAZ7235 - CWR4667		defined
Taxon	Species name including genus and species	categorical	alopecurus ovatus - trisetum spicatum		identified
Trait	Plant functional leaf trait including plant height, wet/dry mass, leaf area, leaf thickness, shoot length, shoot ratio, specific leaf area, leaf dry matter content, water holding capacity, specific root length, carbon, nitrogen and phosphorus content, CN and NP ratio, d13C and d15N isotope ratio	categorical	C_percent - WHC_g_g		defined
Value	Leaf trait value	numeric	−32.999 - 419	cm, g, cm2, mm, cm2/g, percentage, permil	recorded
Elevation_m	Elevation of site	numeric	9.759 - 238.159	m a.s.l.	recorded
Latitude_N	Latitude of site	numeric	78.207 - 78.239	Degree north	recorded
Longitude_E	Longitude of site	numeric	15.34 - 15.712	Degree east	recorded
Functional_group	Plant functional group; vascular and bryophyte	categorical	bryophyte - vascular		identified

The dataset contains 11,345 observations of the covers of 41 taxa in 63 vegetation plots sampled across six sites, three fire histories, and three years. Variable names, description variable type, range or levels, units and short description is given for all variables.

Visual inspection of the unweighted trait distributions indicate that plants influenced by nutrients from a seabird colony are taller and have larger leaves, higher SLA and leaves with lower carbon and higher N and dN^15^ content compared to the reference gradient (Fig. 3b).

The trait datasets from both the ITEX warming experiment and the gradients are suitable for exploring community weighted trait distributions since we have measurements for species making up at least 80% of the cumulative cover for all traits in all plots (calculations based on datasets i). In the warming experiment 96.1% and at the gradient 73.4% of the plots meet this criterion for local (plot-level) trait measurements which makes the data well-suited to study community-level consequences of intraspecific trait variation. Note that due to limited leaf biomass available for chemical analyses, data coverage is lower for traits based on these analyses, for which we have plot-level measurements for species making up 77% of the cumulative cover in the warming experiment and 26% in the gradients.

For an overview of the clean datasets and links to the code to clean and extract these data from the raw data, see Table 1. The final clean data are provided in the “Traits” folder, and the raw data are provided in the “RawData” folder on OSF^66^. The code to download and clean the data can be found in the GitHub repository^67^ in the file R/trait_plan.R.

### Dataset (iv) Soil carbon and nitrogen

The soil carbon and nitrogen dataset from the gradients has 70 observations (gradient × site × plot × variable; Tables 1, 8). There are 21 observations for C and N at the reference gradient and 14 at the nutrient input gradient. Soil carbon or nitrogen content did not vary between the gradients or with elevation.

**Table 8:** Data dictionary for soil carbon and nitrogen (dataset iv) from elevational gradients with and without nutrient input from a seabird colony, in Bjørndalen and on Lindholmhøgda, respectively, near Longyearbyen, Svalbard.

Variable name	Description	Variable type	Variable range or levels	Units	How measured
Gradient	Elevational gradient; C = reference, B = nutrient input gradient (seabird colony)	categorical	B – C		defined
Site	Site as a number 1–7	numeric	1–7		defined
PlotID	Plot ID from A to G	categorical	A–G		defined
Variable	Soil nutrient variable; C = carbon, N = nitrogen	categorical	C–N		defined
Value	Soil nutrient content	numeric	0.096 – 28.388	percentage	recorded
Weight_mg	Weight of soil sample	numeric	3.522 – 8.812	mg	recorded
Elevation_m	Elevation of site	numeric	9.759 – 238.159	m a.s.l.	recorded
Longitude_E	Longitude of site	numeric	15.34 – 15.712	Degree east	recorded
Latitude_N	Latitude of site	numeric	78.207 – 78.239	Degree north	recorded

The dataset contains 70 observations sampled across the two elevational gradients. Variable names, description variable type, range or levels, units and short description is given for all variables.

The average soil carbon content at the nutrient input gradient was 11.6 ± 2.17% and nitrogen content 0.45 ± 0.07%. At the reference gradient, soil carbon content was 6.24 ± 0.77% and nitrogen content 0.243 ± 0.02%, both lower than under the influence of seabirds.

For an overview of the clean datasets and links to the code to clean and extract these data from the raw data, see Table 1. The final clean data are provided in the “Soil” folder, and the raw data are provided in the “RawData” folder on OSF^66^. The code to download and clean the data can be found in the GitHub repository^67^ in the file R/soil_plan.R.

### Dataset (v): Ecosystem CO_2_ flux

The ecosystem CO_2_ flux dataset (NEE, R_eco_ and GPP) from the ITEX warming experiment has 135 individual flux measurements from peak growing season in 2018, paired with their environmental metadata (site × plot × variable; Table 1). Fluxes are generally larger in the dry *Dryas* heath than the wet snowbed community, with the *Cassiope* heath being intermediate. Across the three sites, experimental warming increases both R_eco_ and GPP fluxes, yielding similar NEE across treatments^5^.

For an overview of the datasets and links to the code to clean and extract clean data from the raw data, see Table 1. The raw CO_2_ flux data from both the ITEX warming and gradients is provided as zip files in the “RawData/RawData_C-Flux” folder on OSF^66^. The CO_2_ flux data from the ITEX warming experiment is provided on OSF^66^ as non-standardized raw data “C-Flux/Cflux_SV_ITEX_2018.csv” and as standardized data “C-Flux/Endalen_paper/ITEX_all.Rdata”. For the code to clean and standardize the ITEX flux data, see^5^. Note that we do not provide a clean version of the flux data for the reference and nutrient input gradient.

### Dataset (vi): Remote sensing

In total, we created 5-band orthomosaics, radiometrically calibrated and georeferenced with GCPs, from seven areas, covering 118 ha, built using 28,500 overlapping geolocated images, and with pixel resolutions that range from 2.90 cm to 6.72 cm. We further collected 68 turfs from two sites (ITEX warming experiment and nutrient input gradient) that we used for ground-truthing, and from which we obtained leaf spectroscopy readings and functional traits. These turfs represented 18 species of moss, graminoids, and dwarf shrubs, and generated a total of 340 leaf spectroscopy measurements (spectra). Finally, an additional 117 ground-truthing points were geolocated in the three core sites identified as dwarf shrub, graminoid, or moss (Table 9).

**Table 9:** Summary of the remote sensing data (dataset vi) generated for this paper.

Site name	UAV flights					Spectroscopy/Trait collection		Georeferenced extra ground points	
	Area (ha)	Pixel Resolution (cm)	Latitude (°N)	Longitude (°E)	Num of geolocated images	Single-species turfs for spectroscopy & traits (spectra read)	Num Species measured through field spectroscopy	GNSS vegetation ground truthing points	Num of plant functional groups in ground truthing points
ITEX warming experiment	17,6334	4,58	78,1887	15,7429	4535	23 (115)	3*	37	3
Reference gradient	46,4251	6,08	78,2164	15,6876	10520	—	—	18	3
Nutrient input gradient	20,0827	6,72	78,2410	15,3355	2255	45 (225)	13	62	3
ITEX_ValleyOpposite	7,5274	4,54	78,1844	15,7623	1595	—	—	—	—
Snow Fences	10,0716	3,54	78,1744	16,0577	3750	—	—	—	—
Alluvial Fan	8,1944	2,90	78,1726	16,0354	2910	—	—	—	—
Flux Tower	8,0564	3,50	78,1871	15,9138	2935	—	—	—	—
TOTAL/AVERAGE	117,9910	4,55	—	—	28500	68 (340)	18	117	—

“UAV flights”: main characteristics of the orthomosaics built for each study site. For each flight, radiometrically calibrated reflectance values exist for 5 bands (Red, Green, Blue, Red-Edge, and NIR); *Latitude* and *Longitude* correspond to the origin of the raster (north-west corner); *Num of geolocated images* shows the number of individual overlapping images used to build each orthomosaic. “Spectroscopy/Trait collection”: information on the single-species turfs collected for ground-truthing, leaf spectroscopy, and trait measurements. “Georeferenced extra ground points”: number and functional group of identified vegetation types in the field which were geo-referenced with a differential GNSS system.

*plus 1 moss & 1 graminoid - unidentified

The data are organized in six main categories, namely: (a) Handheld spectra, which contains all the hyperspectral data from the turfs; (b) UAV imagery, which contains the multispectral orthomosaics for each of the sites that were flown; (c) turf species; (d) turf traits; (e) UAV spectra, which contains the multi-spectral information extracted from the orthomosaics for the points where the turfs and ground-truthed species coordinates were taken - read^28^ for further information. A sixth category (f) corresponds to Sentinel imagery used to upscale the maps produced in^28^. A readme text file has been produced for each of these data categories, explaining the metadata in detail.

The remote sensing data can be found on the OSF^66^ repository.

### Dataset (vii): Climate

Climate weather station data from the ITEX warming experiment has a total of 815,339 observations, including air temperature, PAR, relative humidity, water content, and solar radiation data throughout 2015–2018 (date × variable; Tables 1, 10). Average values over the whole period were 153.71 ± 0.64 μmol m^−2^ s^−1^ PAR, 79.11 ± 0.02% relative humidity, 67.14 ± 0.28 W/m² solar radiation, −1.51 ± 0.02 °C and 0.18 ± 0.00 m^3^/m^3^ soil water content. For more details, see^5^.

**Table 10:** Data dictionary for the weather station climate data (dataset vii-a-1) from a climate station at an ITEX warming experiment in Endalen, Svalbard.

Variable name	Description	Variable type	Variable range or levels	Units	How measured
DateTime	Date and time of sampling	date_time	2015-08-13 12:00:00 - 2018-09-18 23:50:00	yyyy-mm-dd	recorded
LoggerLocation	Location of logger; air	categorical	air - air		defined
LoggerType	Logger type; weather station	categorical	WeatherStation - WeatherStation		defined
Variable	Climate variable; PAR, water content, air temperature, relative humidity, and solar radiation	categorical	PAR - WaterContent		defined
Value	Climate variable value	numeric	−23.835 - 2048.7	umol m-2 s-1, m3 m-3, degree celsius, percentage, W m-2	recorded

The dataset contains three years of data for temperature, PAR, relative humidity, water content, and solar radiation. Variable names, description variable type, range or levels, units and short description is given for all variables.

Temperature logger data from the ITEX warming experiment has a total of 937,388 observations of soil and surface temperatures from 2004–2005 and 2015–2018 (date × site × treatment × logger; Tables 1, 11).

**Table 11:** Data dictionary for the climate logger data (dataset vii-a-2) from an ITEX warming experiment in Endalen, Svalbard.

Variable name	Description	Variable type	Variable range or levels	Units	How measured
DateTime	Date and time of sampling	date_time	2004-09-03 16:00:29 - 2018-07-10 16:02:01	yyyy-mm-dd	recorded
Site	Site as the habitat; DH = Dryas heath, CH = Cassiope heath and SB = snowbed	categorical	CH - SB		defined
Treatment	Warming treatment; CTL = ambient conditions, OTC = warmed by Open top chamber (OTC)	categorical	CTL - OTC		defined
PlotID	Unique ID as the combination of Site and number; SB-1	categorical	CH-1 - SB-9		defined
LoggerType	Logger type; iButton and Tiny Tag	categorical	iButton - TinyTag		defined
LoggerLocation	Location of logger; surface and soil	categorical	soil - surface		defined
Variable	Climate variable; surface and soil temperature	categorical	Temperature - Temperature		defined
Value	Temperature value	numeric	−27.35 - 54.062	Degree celsius	recorded

The dataset contains climate logger observations sampled across three habitats between 2004 and 2018. Variable names, description variable type, range or levels, units and short description is given for all variables.

The mean summer ground surface temperature (June–September) in the periods between 2004–2005 and 2015–2018 was 7.7 ± 0.01 °C and the soil temperature was 5.37 ± 0.01 (dataset vi-a-1, Table 11). The OTCs increase the summer ground surface temperature by between 0.62–1.67 °C and the soil temperature by 0.49–1.03 °C, except for in the *Cassiope* heath where the temperature was −0.70 °C colder in the OTC compared to the control plots in this period.

Climate data from the gradient has a total of 162 observations (n = 81 for soil moisture and temperature each; gradient × site × plot × variable; Tables 1, 12). Soil temperature was higher at the reference gradient (E = 2.68, t_71_ = 6.11, P < 0.001). Soil moisture decreased with elevation but more strongly at the nutrient input gradient (E = −0.10, t71 = −3.42, P = 0.001).

**Table 12:** Data dictionary for the climate data (dataset vii-b) from elevational gradients with and without marine nutrient input from a seabird colony, in Bjørndalen and on Lindholmhøgda, respectively, near Longyearbyen, Svalbard.

Variable name	Description	Variable type	Variable range or levels	Units	How measured
Year	Year of sampling	numeric	2018 - 2018	yyyy	recorded
LoggerType	Type of logger; iButton	categorical	iButton - iButton		defined
LoggerLocation	Location of logger; soil	categorical	soil - soil		defined
Gradient	Elevational gradient; reference or nutrient input gradient (seabird colony)	categorical	B - C		defined
Site	Site as a number 1–7	categorical	1–7		defined
PlotID	Plot ID from A to G	categorical	A - G		defined
Variable	Climate variable; soil temperature and soil moisture	categorical	SoilMoisture - SoilTemperature		defined
Value	Climate value	numeric	3.6–55.1	Degree celsius, (m3 water × m-3 soil) × 100	recorded

The dataset contains 70 observations sampled across two elevational gradients. Variable names, description variable type, range or levels, units and short description is given for all variables.

For an overview of the clean datasets and links to the code to clean and extract these data from the raw data, see Table 1. The final clean data are provided in the “Climate” folder, and the raw data are provided in the “RawData” folder on OSF^66^. The code to download and clean the data can be found in the GitHub repository^67^ in the file R/climate_plan.R.

## Technical Validation

### Taxonomic validation

We took vouchers from all taxa, and Pernille Bronken Eidessen (UNIS) and other local experts checked taxonomic identification. We identified the species to the lowest taxonomic level possible, but in some cases, the taxonomy changed during the course of the 17-year study, and we were not always able to distinguish closely related taxa, such as for example within *Poa pratensis* (see discussion in https://www.svalbardflora.no/). Specimens that were unidentifiable to species in the field were given a descriptive name, and the voucher was stored. The community data thus has 19 unidentified specimens where only the genus is known, and one completely unknown specimen. Fifteen of those are lichen and bryophytes from the ITEX experiment, and the other four are forbs and graminoids from the gradients (dataset i). There are no unidentified taxa in the trait data (dataset iii). The final community taxonomy and trait data were checked and corrected against the Taxonomic Nomenclature Resolution Service (TNRS)^48,49^ (see above). Note that for some common taxa on Svalbard, such as *Festuca richardsonii*, there is a discrepancy between the TNRS accepted name and the name used in the current Svalbardflora (https://www.svalbardflora.no/). For clarity, we refer to these taxa by their TNRS names in the text. A full species list of all identified species, including their authority across datasets, is also available in the OSF repository^66^ in the ‘Community’ folder (PFTC4_Svalbard_2018_Species_list.csv).

### Community data validation

We checked and corrected missing or unrealistic cover values against the field notes for typing errors. The data-checking and outcomes of correction procedures is documented in the code^67^.

### Trait data validation

Trait data were thoroughly checked and validated as follows. First, we checked and corrected missing or erroneous sample identifications in one or more measurements against field notes and notes on the leaf envelopes. Second, unrealistically high or low values of one or more trait values were checked and corrected against the lab and field notes for typing errors, and/or leaf scans were checked for problems during the scanning process (e.g., empty scans, double scans, blank areas within the leaf perimeter, dirt, or other non-leaf objects on scans). Issues that could be resolved were corrected (e.g., recalculating the leaf area manually to include missing leaf parts on the scan, the wrong match between scan and leaf ID, etc.). Any remaining samples with apparent measurement errors that resulted in unrealistic trait values were removed, as follows: Leaves with specific leaf area values greater than 500 cm^2^ g^−1^ were removed (n = 76). Because it was difficult to find out why the SLA values were so high, the dry mass and leaf area values of those leaves were also removed. Leaves with carbon values higher than 6.4% (n = 8) and phosphorus values higher than 5% (n = 25) were deemed unrealistic and also removed. See the code^67^ for details. We further plotted the data (e.g., wet mass vs. dry mass) and checked for outliers. The data checking and outcomes for these various correction procedures are available and documented in the code^67^ and associated readme file.

### Ecosystem CO_2_ flux validation

Each flux measurement curve was assessed visually for quality. We checked for inconsistencies within the data of each measurement by plotting CO_2_ air concentration vs. time. The linearity of CO_2_ increase/decrease was assessed by *r*^2^ values of linear regression models. Time-intervals used for calculations were adjusted manually if inconsistencies occurred (e.g., due to outliers or signs of leakage).

### Remote sensing data validation

Remote sensing data were collected using best-practice and thoroughly checked and validated. The UAV was flown with a MicaSense RedEdge Downwelling Light Sensor (DLS). The imagery was radiometrically calibrated using a MicaSense reflectance panel as the calibration target. Ground control points for georeferencing were taken using the Emlid Reach + differential GNSS system (Emlid, Hong Kong). RMS errors for all orthomosaics were <0.2 m. Pix4D quality reports ensured the images were stitched to the highest possible quality and accuracy.

After every turf measurement, the field spectroradiometer was optimized and calibrated for dark current and white light a. Each measurement consisted of 40 internally averaged reflectance readings to increase the signal-to-noise ratio. The spectra were visually assessed to ensure there were no bad measurements. Turf traits values were checked for unrealistically high or low values as described above.

### Climate data validation

The climate data of each plot was visually inspected, and unrealistic high or low values were removed.

## Usage Notes

To properly use these data, be aware that: (a) The community data contains a few unidentified specimens, and for some taxonomic groups, especially within *Poa*, identifications may be uncertain (see above, and comments or flags in the raw data^66^). (b) The ITEX experiment community data (dataset i) contains two control plots and two OTC plots in the *Cassiope* heath with icing damage, flagged with “iced” in the “Flag” column, that should be removed from the dataset to avoid bias in the species richness and abundance of the *Cassiope*habitat due to these plots. (c) In the height data from the ITEX warming experiment, vegetation height was measured differently in 2009 and 2015 and while both methods are valid the measurements cannot be directly compared. (d) In the traits data we have followed what we consider best practice for data quality and filtered out what we consider unreliable data, e.g., dry mass for very small leaves approaching the limits for balance accuracy, leaf area in the case of clearly erroneous scans, and some clearly unrealistic measurements or calculations (see above). (e) The trait data from the ITEX warming experiment contains leaves from one *Betula nana* individual. This plant is not part of the ITEX experiment and was found near the study site. This is the only *Betula nana* individual we encountered during the field work, and VV could not be restrained from collecting a few leaves. During data cleaning, AHH could not bear to remove these precious leaves from the dataset, and all authors agreed they deserved to live on as an electronic legacy of these events. Therefore, we rely on the user’s responsibility to read this usage note and remove this species from their analysis as necessary. (f) The slopes in the ecosystem CO_2_ flux data from the ITEX experiment were calculated using a linear model^5^, and we provide the raw data on OSF^66^ if users want to use a different method. The flux data should be standardized by temperature, PAR and/or biomass estimates before being used. For an example of how to do this, see^5^. We only provide raw flux data from the gradients.

Note that the ITEX site is part of the larger ITEX community (https://www.gvsu.edu/itex/) and some of the data reported here along with additional data from the site are part of a community database within this network, and also of scientific publications within the ITEX community.

The two elevational gradients are designed to be comparable (e.g. similar bedrock, grazing regime, andmicrohabitat) and comparative studies are encouraged. However, there are topographical differences (e.g. slope, microtopography,microclimate), which should be taken into consideration when comparing the two gradients, see^28^.

### Data and terminology

Note that the nutrient input gradient is coded as ‘B’ in all datasets, referring to the general impact from sea*bird* colonies or *bird*-cliffs (although note that in Bjørndalen, the little auk nest in the ground on the upper part of the talus slope below the cliff, so this is a colony, not a bird-cliff). In this paper, we use the terminology ‘nutrient input gradient’ as this focus on nutrients, not birds *per se*, is the most relevant from the plant’s point of view. We did not change the terminology in the data, however, as these have already been used in publications^5,28^. Also, note that species names are lower case variables in the data, and will need to be corrected (capitalize genus names, use italics) for usage in text and figures.

For all datasets, see the code^67^ for our suggested data cleaning and checking procedures that result in producing what we consider the clean and ‘best practice’ final datasets and the various ‘Flag’ and ‘Comment’ columns in the different dataset tables that indicate additional specific data points or individual observations (e.g., leaves, data rows) that could be removed to create even more robust datasets.

## Data Availability

The code used for checking, cleaning, and analyzing the data is available in the open GitHub repository “https://github.com/Plant-Functional-Trait-Course/PFTC_4_Svalbard”, of which a versioned copy is available at Zenodo^67^.

## References

[1] Rantanen M (2022). The Arctic has warmed nearly four times faster than the globe since 1979. Communications Earth & Environment.

[2] IPCC. *Climate Change 2021: The Physical Science Basis. Contribution of Working Group I to the Sixth Assessment Report of the Intergovernmental Panel on Climate Change* (ed. Masson-Delmotte, V., *et al* (Cambridge University Press, 2021).

[3] Paleczny M, Hammill E, Karpouzi V, Pauly D (2015). Population Trend of the World’s Monitored Seabirds, 1950–2010. PLoS One.

[4] Dias MP (2019). Threats to seabirds: A global assessment. Biol. Conserv..

[5] Jónsdóttir IS (2022). Intraspecific trait variability is a key feature underlying high Arctic plant community resistance to climate warming. Ecol. Monogr..

[6] Myers-Smith IH (2020). Complexity revealed in the greening of the Arctic. Nat. Clim. Chang..

[7] Elmendorf SC (2012). Global assessment of experimental climate warming on tundra vegetation: heterogeneity over space and time. Ecol. Lett..

[8] Urban MC (2016). Improving the forecast for biodiversity under climate change. Science.

[9] Mekonnen ZA (2021). Arctic tundra shrubification: a review of mechanisms and impacts on ecosystem carbon balance. Environ. Res. Lett..

[10] Bjorkman AD (2018). Plant functional trait change across a warming tundra biome. Nature.

[11] Bjorkman AD (2019). Status and trends in Arctic vegetation: Evidence from experimental warming and long-term monitoring. Ambio.

[12] Treharne R, Bjerke JW, Tømmervik H, Stendardi L, Phoenix GK (2019). Arctic browning: Impacts of extreme climatic events on heathland ecosystem CO_2_ fluxes. Glob. Chang. Biol..

[13] Grant ML, Bond AL, Lavers JL (2022). The influence of seabirds on their breeding, roosting and nesting grounds: A systematic review and meta-analysis. J. Anim. Ecol..

[14] Caut S (2012). Seabird modulations of isotopic nitrogen on islands. PLoS One.

[15] Benkwitt CE, Carr P, Wilson SK, Graham NAJ (2022). Seabird diversity and biomass enhance cross-ecosystem nutrient subsidies. Proc. Biol. Sci..

[16] Lorrain A (2017). Seabirds supply nitrogen to reef-building corals on remote Pacific islets. Sci. Rep..

[17] Earl JE, Zollner PA (2017). Advancing research on animal-transported subsidies by integrating animal movement and ecosystem modelling. J. Anim. Ecol..

[18] De La Peña-Lastra S (2021). Seabird droppings: Effects on a global and local level. Sci. Total Environ..

[19] Zwolicki A, Zmudczyńska-Skarbek KM, Iliszko L, Stempniewicz L (2013). Guano deposition and nutrient enrichment in the vicinity of planktivorous and piscivorous seabird colonies in Spitsbergen. Polar Biol..

[20] García Criado M (2023). Plant traits poorly predict winner and loser shrub species in a warming tundra biome. Nat. Commun..

[21] Kemppinen J (2021). Consistent trait-environment relationships within and across tundra plant communities. Nat. Ecol. Evol..

[22] Hamann E, Kesselring H, Stöcklin J (2018). Plant responses to simulated warming and drought: a comparative study of functional plasticity between congeneric mid and high elevation species. J Plant Ecol.

[23] Henn JJ (2018). Intraspecific Trait Variation and Phenotypic Plasticity Mediate Alpine Plant Species Response to Climate Change. Front. Plant Sci..

[24] Siefert A (2015). A global meta-analysis of the relative extent of intraspecific trait variation in plant communities. Ecol. Lett..

[25] Funk JL (2017). Revisiting the Holy Grail: using plant functional traits to understand ecological processes. Biol. Rev. Camb. Philos. Soc..

[26] Lavorel S, Garnier E (2002). Predicting changes in community composition and ecosystem functioning from plant traits: revisiting the Holy Grail. Funct. Ecol..

[27] Chacón-Labella J (2023). How to improve scaling from traits to ecosystem processes. Trends Ecol. Evol..

[28] Thomson ER (2021). Multiscale mapping of plant functional groups and plant traits in the High Arctic using field spectroscopy, UAV imagery and Sentinel-2A data. Environ. Res. Lett..

[29] Schweiger AK (2017). How to predict plant functional types using imaging spectroscopy: linking vegetation community traits, plant functional types and spectral response. Methods Ecol. Evol..

[30] Cavender-Bares J (2022). Integrating remote sensing with ecology and evolution to advance biodiversity conservation. Nat Ecol Evol.

[31] Dietze MC, Lebauer DS, Kooper R (2013). On improving the communication between models and data. Plant Cell Environ..

[32] Kattge J (2020). TRY plant trait database - enhanced coverage and open access. Glob. Chang. Biol..

[33] Patrick L, Thompson S, Halbritter AH (2020). Adding value to a field‐based course with a science communication module on local perceptions of climate change. Bulletin of the Ecological Society of America.

[34] Geange SR (2020). Next generation field courses: integrating Open Science and online learning. Ecol. Evol..

[35] Vandvik V (2020). Plant traits and vegetation data from climate warming experiments along an 1100 m elevation gradient in Gongga Mountains, China. Sci Data.

[36] Halbritter AH (2023). Zenodo.

[37] Halbritter AH (2020). The handbook for standardized field and laboratory measurements in terrestrial climate change experiments and observational studies (ClimEx). Methods Ecol. Evol..

[38] Wilkinson MD (2016). The FAIR Guiding Principles for scientific data management and stewardship. Sci Data.

[39] Alston JM, Rick JA (2021). A beginner’s guide to conducting reproducible research. Bull. Ecol. Soc. Am..

[40] Hampton SE (2015). The Tao of open science for ecology. Ecosphere.

[41] Vandvik V (2022). The role of plant functional groups mediating climate impacts on carbon and biodiversity of alpine grasslands. Sci Data.

[42] Jones, A. *et al*. *Soil atlas of the northern Circumpolar Region*. (European Commission, 2010).

[43] Jónsdóttir, I. S. International Tundra Experiment ITEX - Expert Network Monitoring Plan. Supporting publication to the CAFF Circumpolar Biodiversity Monitoring Program Framework Document. **8** (2004).

[44] Henry GHR, Molau U (1997). Tundra plants and climate change: the International Tundra Experiment (ITEX). Glob. Chang. Biol..

[45] Dallmann, W. K. Geoscience Atlas of Svalbard. (2015).

[46] Pirk N (2017). Spatial variability of CO_2_ uptake in polygonal tundra: assessing low-frequency disturbances in eddy covariance flux estimates. Biogeosciences.

[47] Cooper EJ, Little CJ, Pilsbacher AK, Mörsdorf MA (2019). Disappearing green: Shrubs decline and bryophytes increase with nine years of increased snow accumulation in the High Arctic. J. Veg. Sci..

[48] Maitner, B. & Boyle, B. TNRS: Taxonomic Name Resolution Service. Preprint at https://CRAN.R-project.org/package=TNRS (2021).

[49] Boyle B (2013). The taxonomic name resolution service: an online tool for automated standardization of plant names. BMC Bioinformatics.

[50] Missouri Botanical Garden. Tropicos. Preprint at http://www.tropicos.org (2012).

[51] TPL. The plant list version 1.1. Preprint at http://www.theplantlist.org, (2013).

[52] USDA, NRCS. The PLANTS Database. Preprint at http://plants.usda.gov (2015).

[53] Hallingbäck, T., Lönnell, N., Weibull, H. & Hedenäs, L. *Bladmossor: Sköldmossor - blåmossor: Bryophyta: Buxbaumia – Leucobryum*. (SLU Artdatabanken, 2005).

[54] Molau, U. & Mølgaard, P. International tundra experiment (ITEX) manual. *Danish Polar Center, Copenhagen, Denmark* (1996).

[55] Pérez-Harguindeguy N (2013). New handbook for standardised measurement of plant functional traits worldwide. Austr. J. Bot..

[56] Roos RE (2019). Contrasting drivers of community‐level trait variation for vascular plants, lichens and bryophytes across an elevational gradient. Funct. Ecol..

[57] van Zuijlen K (2022). Community-level functional traits of alpine vascular plants, bryophytes, and lichens after long-term experimental warming. Arct. Sci..

[58] Schneider CA, Rasband WS, Eliceiri KW (2012). NIH Image to ImageJ: 25 years of image analysis. Nat. Methods.

[59] Katabuchi, M. *LeafArea: Rapid Digital Image Analysis of Leaf Area*. (2017).

[60] Matejovic I (1997). Determination of carbon and nitrogen in samples of various soils by the dry combustion. Commun. Soil Sci. Plant Anal..

[61] Jasoni, R. L., Smith, S. D. & Arnone, J. A. III Net ecosystem CO_2_ exchange in Mojave Desert shrublands during the eighth year of exposure to elevated CO_2_. *Glob. Chang. Biol*. (2005).

[62] Street, L. E., Shaver, G. R. & Williams, M. What is the relationship between changes in canopy leaf area and changes in photosynthetic CO_2_ flux in arctic ecosystems? *Journal of* (2007).

[63] Huxman TE (2004). Precipitation pulses and carbon fluxes in semiarid and arid ecosystems. Oecologia.

[64] Potts D. L. *et al*. Antecedent moisture and seasonal precipitation influence the response of canopy-scale carbon and water exchange to rainfall pulses in a semi-arid grassland. *New. Phyt*. **70**, 849–860. 10.1111/j.1469-8137.2006.01732.x 16684243

[65] Bjerke JW (2017). Understanding the drivers of extensive plant damage in boreal and Arctic ecosystems: Insights from field surveys in the aftermath of damage. Sci. Total Environ..

[66] Halbritter AH (2023). OSF.

[67] Halbritter AH (2023). Zenodo.

[68] CASRAI. *CRediT - Contributor Roles Taxonomy*. Retrieved from https://casrai.org/credit/ (2019).

